# Preliminary findings from stimulated spontaneous reporting of adverse drug reactions during COVID-19 pandemic: an experience from Ghana

**DOI:** 10.4314/gmj.v54i4s.10

**Published:** 2020-12

**Authors:** Delese A Darko, Seth K Seaneke, George T Sabblah, Adela Ashie, Abena Asamoa-Amoakohene, Jeremiah S Ewudzie, Theodora Asa-Eck, Ernest Agyei-Kwame, Felicia Dwamena, Josephine Mensah, Jennifer Boateng

**Affiliations:** 1 Food and Drugs Authority, P. O. Box CT 2783, Accra; 2 University of Ghana Medical Centre, Post Office Box LG 25, Legon, Accra; 3 Greater Accra Regional Hospital, P. O. Box GP 473, Accra

**Keywords:** adverse drug reaction, spontaneous reporting, COVID-19, pandemic

## Abstract

**Background:**

The novel coronavirus disease 2019 (COVID-19) is an ongoing pandemic caused by severe acute respiratory syndrome coronavirus 2 (SARS-CoV-2). There is limited information on the safety of drugs used for the treatment of COVID-19.

**Objective:**

Objective of this study is to describe the pattern of stimulated spontaneous adverse drug reaction (ADR) reports received from healthcare professionals for SARS-CoV-2 positive patients in Ghana and lessons learnt particularly for low- and middle-income countries.

**Methods:**

This is a study of individual case safety reports (ICSRs) received from healthcare professionals between 1^st^ April 2020 to 31^st^ July 2020 in SARS-CoV-2 positive patients in Ghana. The ICSRs were retrieved from the SafetyWatch System and descriptive statistics used to describe the ADRs by System Organ Classification and Preferred Term.

**Results:**

Information was received from 40 COVID-19 Treatment Centres across the country with 9 centres submitting a total of 53 ICSRs containing 101 ADRs; approximately two ADRs per ICSR. Females accounted for 29(54.7%) of the ICSRs and males 24(45.3%). Newly reported ADRs of interest were one report each of tremor for doxycycline; scrotal pain, dyspnoea, gait disturbances and dysgeusia for chloroquine; and dry throat, hyperhidrosis, restlessness and micturition frequency increased for hydroxychloroquine. A strong spontaneous system with the availability of focal persons at the Treatment Centres played a key role in reporting ADRs during the pandemic.

**Conclusion:**

This is the first experience with spontaneous reporting during COVID-19 pandemic in Ghana. The profile of most of the ADRs reported appears consistent with what is expected from the summary of product characteristics. A study with a larger sample size with well-defined denominator in future studies is paramount in determining the relative risk of these medications in SARS-CoV-2 positive patients.

**Funding:**

None declared

## Introduction

The novel coronavirus disease 2019 (COVID-19) is an ongoing pandemic caused by severe acute respiratory syndrome coronavirus 2 (SARS-CoV-2). The outbreak was first identified in Wuhan, China, in December 2019.^1^ The World Health Organization declared the outbreak a Public Health Emergency of International Concern on 30^th^ January 2020^2^ and then a pandemic on 11^th^ March 2020.^3^ Ghana recorded the first two cases of COVID-19 on 12^th^ March 2020 and as at 27^th^ July 2020 there were 35,142 confirmed cases in Ghana with 175 deaths.^4^

Remdesivir is the only therapeutic agent approved at the moment for the treatment of COVID-19 by the U. S. Food and Drugs Administration (FDA) and the European Medicine Agency (EMA) based on results from randomised clinical trials.^5^,^6^,^7^ The European Medicine Agency is currently reviewing dexamethasone for possible approval based on the results from the RECOVERY trial.^5^,^8^ There are ongoing studies to find effective therapeutics and vaccines for COVID-19.^9^

In Ghana, the Ministry of Health has developed the Provisional Standard Treatment Guidelines for Novel Coronavirus Infection to use repurposed medicines, namely hydroxychloroquine, chloroquine, azithromycin, doxycycline, methylprednisolone, tocilizumab and remdesivir (if available) for the treatment of patients with COVID-19 based on emerging data from various studies including clinical trials as well as existing standard treatments for supportive care.^10^ The drugs for the treatment of COVID-19 were approved in line with the Ghana FDA's Guidelines for Emergency Use Authorization of Medical Products which authorises the FDA to assess and list unregistered medical product in consultation with the Ministry of Health for use primarily during public health emergencies of international concern.^11^ These products and their uses are not approved, cleared, or registered under section 118 of the Public Health Act (Act 851).^12^ The FDA guideline also requires that safety and effectiveness data are collated during the period of the emergency use authorisation to inform the continuous use of these products.^11^ There are also a number of drugs for supportive treatment using existing therapies based on the 7^th^ edition of the 2017 Standard Treatment Guidelines.^13^

Although, the drugs issued with emergency use authorisation except remdesivir have been approved and used in Ghana for other disease conditions, none has been used in the context of COVID-19, so, there is lack of information on the effect of these drugs in the treatment of this disease. There are also concerns worldwide about cardiac adverse drug reactions with potential drugs for the treatment of COVID-19^14^, particularly chloroquine or hydroxychloroquine alone or in combination with azithromycin. A French study found that of adverse drug reactions received for the use of hydroxychloroquine, azithromycin, lopinavir-ritonavir and chloroquine in COVID-19 within a month, 120 reports of cardiac adverse drug reactions were received for which 103(86%) were associated with hydroxychloroquine and 60% with azithromycin,^15^ it was however not clear if these products were administered alone or in combination with other drugs.

A Cochrane review by the World Health Organization revealed that the current evidence on the safety and efficacy of hydroxychloroquine for the treatment of COVID-19 is limited and of very low certainty.^16^ The hydroxychloroquine arms of the RECOVERY and SOLIDARITY trials have been suspended^17^,^18^ and countries like the United States of America and France have stopped the use of hydroxychloroquine in COVID-19.^19^,^20^ At the time of writing this paper, hydroxychloroquine and chloroquine are being used in lower-middle income countries, including, India, Brazil and Ghana for COVID-19. 10,21,22

There is, therefore, the need to gather some data on the safety of the use of these products to inform their continuous use during the pandemic. Additionally, there is limited information on the safety of other drugs used together with those issued with emergency use authorisation in the context of COVID-19. The objective of this study is to describe the pattern of spontaneous adverse drug reaction (ADR) reports received from healthcare professionals in patients who tested positive for SARSCoV-2 in Ghana and the lessons learnt particularly for low- and middle-income countries.

## Methods

### Study design

The study was a prospective study designed to receive individual case safety reports (ICSRs) from healthcare professionals through a stimulated spontaneous reporting system between 1^st^ April 2020 to 31^st^ July 2020 in patients who tested positive for SARS-CoV-2 in Ghana and on treatment with any medication.

### Data collection and settings

Ghana joined the WHO Programme for International Drug Monitoring since November 2001 with the Food and Drugs Authority designated as the National Pharmacovigilance Centre. The National Pharmacovigilance Centre operates a safety monitoring system which relies on spontaneous reporting from healthcare professionals and focal points at the healthcare facilities called the Institutional Contact Persons. In this study, the Institutional Contact Persons and the FDA Regional Offices were instrumental in the collation of ADR reports from healthcare facilities designated by the Ministry of Health as COVID-19 Treatment Centres through a stimulated spontaneous reporting system. Stimulated spontaneous reporting is a type of spontaneous reporting method used to encourage and facilitate reporting by health professionals for new products, or the limited period.

Before the study, the National Pharmacovigilance Centre developed the Safety Monitoring Plan for Medicines for the Treatment of COVID-19 which was shared with the Heads of the Treatment Centres, COVID-19 Case Management Teams and the Institutional Contact Persons. The objective of the Safety Monitoring Plan is to outline the procedures for stimulated pharmacovigilance during the COVID-19 pandemic to ensure that healthcare professionals report all suspected adverse drug reactions associated with medicines issued with Emergency Use Authorization and all other medicines used for the treatment of patients diagnosed with COVID-19. The plan also outlines when, how, and what to report and the timelines for reporting.

There were daily follow-ups and reminders to the Institutional Contact Persons on the need to follow-up on patients being treated for COVID-19 and report any ADRs including therapeutic failures. The Institutional Contact Persons were also required to submit weekly updates to the National Pharmacovigilance Centre.

The ICSRs were retrieved from the SafetyWatch System, the Ghanaian pharmacovigilance database which stores ICSRs for medicines (including herbal products) and vaccines. ICSRs in the SafetyWatch System are received from healthcare professionals, marketing authorisation holders and patients.

The SafetyWatch System uses Medical Dictionary for Regulatory Activities (MedDRA)^23^ Version 21.0 for coding ADRs. All reactions were coded using the Preferred Terms (PTs). Information retrieved from the Safety-Watch System on each ICSRs were, the generic name of the suspected and concomitant medications, the age and sex of the patient, description of the adverse drug reaction, seriousness criteria, causality assessment, the date of administration of the suspected drug, dates of onset and stop of the ADR and the outcome of the ADR. The seriousness of the ADRs was based on the Council for International Organizations of Medical Sciences definition.^24^

### Causality assessment

Causality assessment is the evaluation of the relationship between drug treatment and the occurrence of an ADRs. It is also used to evaluate and to check that the particular treatment is the cause of an observed adverse drug reaction or not and to estimate the strength of the relationship between drug(s) exposure and occurrence of ADRs.

Causality assessment of ADR reports received done by the Food and Drugs Authority's Technical Advisory Committee on Safety of Medicines (TAC-SM) using the WHO-UMC system for standardised case causality assessment.^25^ The TAC-SM routinely performs causality assessment for all ADRs received by the FDA on a bimonthly basis. The members of TAC-SM review each ADR taking into consideration the temporal relationship between the drug administration and onset of reaction, individual patient factors including comorbid conditions and concomitant medicines as well as the pharmacological properties of the suspected drug(s).

Expectedness of each adverse drug reaction was assessed using the Summary of Product Characteristics (SmPCs). Time to onset was the number of days from the administration of the suspected drug to the onset of reported ADR(s).

### Ethical approval

According to the present Standard Operating Procedure of the Ghana Health Service Ethics Review Committee, ethical approval is not deemed necessary for this study.^26^

## Results

### Description of ADRs

Information was received from 40 COVID-19 Treatment Centres in all the 16 regions of Ghana with 9 centres in 9 regions submitting a total of 53 ICSRs. Females accounted for 29(54.7%) of the ICSRs and males 24(45.3%). The mean age was 37.8 years (range 4–80 years).

Of the 53 ICSRs received, there were a total of 101 ADRs (approximately two ADRs per ICSR) classified by the Preferred Terms (PTs) using MedDRA version 21.0. Except for seven ADRs classified by PTs as Treatment failure, circumstance or information capable of leading to medication error and Medication error, 85(90.4%) of the ADRs were expected as per the Summary of the Product Characteristics, and the remaining 9(9.6%) were not. Newly reported ADRs of interest were one report each of tremor for doxycycline; scrotal pain, dyspnoea, gait disturbances and dysgeusia reported for chloroquine; and dry throat, hyperhidrosis, restlessness and micturition frequency increased for hydroxychloroquine.

### Description of serious ADRs

Of the 53 ICSRs received during the study period, 3 (5.7%) were serious (life-threatening) and the rest nonserious. The first serious ICSR was received from a 26-year-old male COVID-19 patient who was given azithromycin and hydroxychloroquine and on the same day (17^th^ April 2020), the patient had supraventricular tachycardia, chest discomfort and abdominal pain which all started the same day the suspected drugs were administered.

Concomitant drugs were started the same day as the suspected drugs were zinc, enoxaparin injection, vitamin C and ceftriaxone. Table 1 shows the distribution of ADRs of the primary System Organ Classification (SOC) in decreasing frequency. Gastrointestinal disorders, nervous system disorders and general disorders and administration site conditions were the three most frequently reported SOCs.

**Table 1:** Description of ADRs and type of drugs

ADR (Classified as SOC and PT)	No. of ADRs (%)	Suspected Drugs (Number of ADRs)
**Gastrointestinal disorders**	23 (22.8)	Hydroxychloroquine (6), Aminophylline/Magnesium sulphate (3), Hydroxychloroquine/ Azithromycin (3), Chloroquine (4), Azithromycin (1), Vitamin C (1), Lopinavir/Ritonavir (1), Azithromycin (2), Metformin/Vildagliptin (1)
**Diarrhoea**	13	
**Abdominal pain**	1	
**Abdominal pain upper**	2	
**Nausea**	2	
**Abdominal discomfort**	2	
**Vomiting**	1	
**Oropharyngeal pain**	1	
**Nervous system disorders**	17 (16.8)	Hydroxychloroquine (7), Chloroquine (5), Azithromycin (2), Doxycycline (1), Hydroxychloroquine/Azithromycin (2), Aminophylline/Magnesium sulphate (1)
**Dizziness**	7	
**Headache**	5	
**Tremor**	3	
**Restlessness**	1	
**Dysgeusia**	1	
**General disorders and administration site conditions**	14 (13.9)	Hydroxychloroquine (7), Chloroquine (5), Meropenem (1), Zinc (1)
**Asthenia**	4	
**Treatment failure**	3	
**Pyrexia**	2	
**Lethargy**	1	
**Hyperhidrosis**	1	
**Weak joint**	1	
**Gait disturbances**	1	
**Fatigue**	1	
**Cardiac disorders**	11 (10.9)	Hydroxychloroquine (5), Hydroxychloroquine/Azithromycin (3), Chloroquine (1), Aminophylline/Magnesium sulphate (1), Azithromycin (1)
**Palpitations**	5	
**Sinus tachycardia**	2	
**Supraventricular tachycardia**	1	
**Chest pain**	1	
**Chest discomfort**	1	
**Electrocardiogram QT prolonged**	1	
**ADR (Classified as SOC and PT)**	**Number of Cases**	**Suspected Drugs (Number of cases)**
**Psychiatric disorders**	9 (8.9)	Hydroxychloroquine (6), Chloroquine (2), Hydroxychloroquine/Azithromycin (1)
**Insomnia**	4	
**Hallucinations**	2	
**Somnolence**	1	
**Mood swings**	1	
**Slurred speech**	1	
**Skin and subcutaneous tissue disorders**	9 (8.9)	Hydroxychloroquine (2), Chloroquine (2), Ciprofloxacin (2), Azithromycin (1), Tramadol/Paracetamol (1), Amoxicillin / Clavulanic acid (1)
**Pruritus**	6	
**Rash**	2	
**Urticaria**	1	
**Respiratory, thoracic and mediastinal disorders**	7 (6.9)	Hydroxychloroquine/Azithromycin (3), Hydroxychloroquine (1), Chloroquine (1), Azithromycin (1), Aminophylline/Magnesium sulphate (1)
**Dyspnoea**	5	
**Dry throat**	1	
**Cough**	1	
**Eye disorders**	3 (3.0)	Hydroxychloroquine (2), Chloroquine (1)
**Vision blurred**	2	
**Eye pain**	1	
**Injury, poisoning and procedural complications**	4 (4.0)	Chloroquine (1), Azithromycin (1), Amiodarone (1), Esomeprazole (1)
**Circumstance or information capable of leading to medication** **error**	1	
**Medication error**	3	
**Renal and urinary disorders**	2 (2.0)	Hydroxychloroquine/Azithromycin (1), Chloroquine (1)
**Chromaturia**	2	
**Reproductive system and breast disorders**	1 (1.0)	Hydroxychloroquine (2)
**Scrotal pain**	1	
**Metabolism and nutrition disorders**	1 (1.0)	Chloroquine (1)
**Micturition frequency increased**	1	

**Figure 1:**
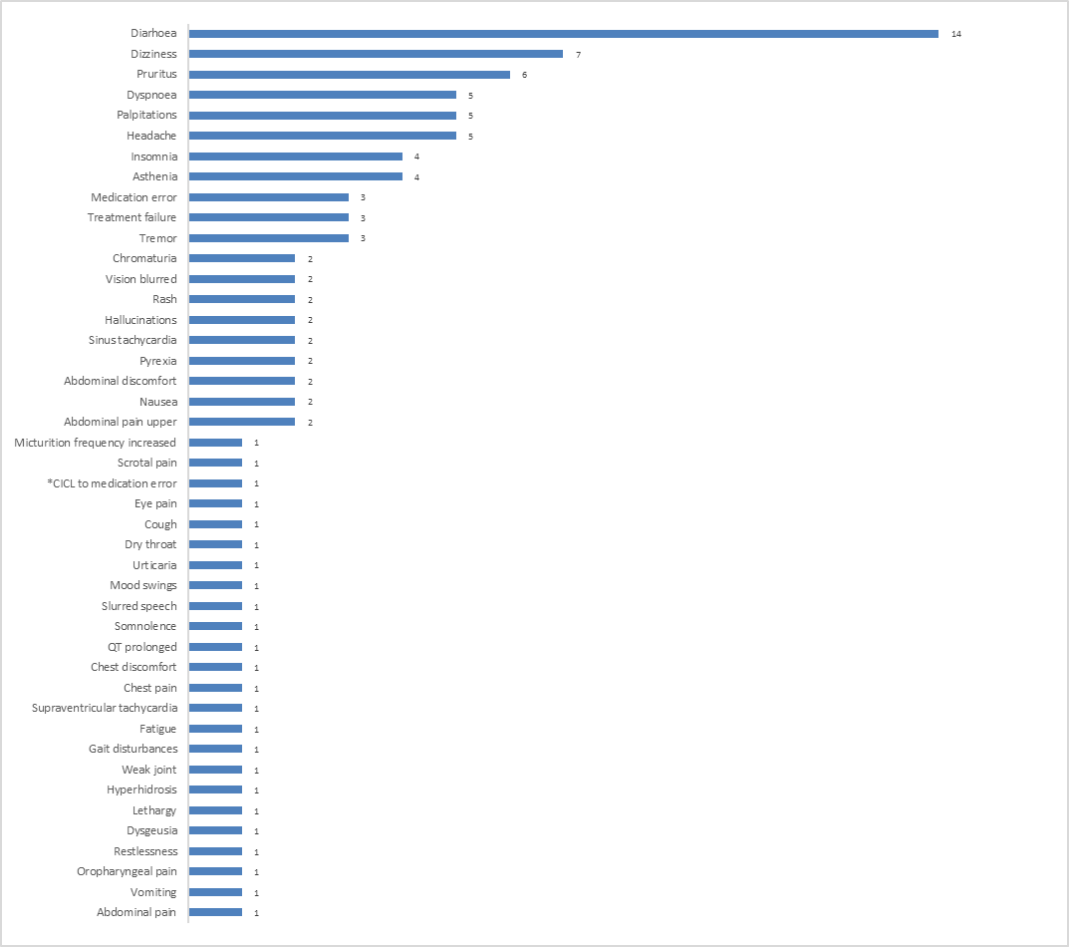
Frequency of adverse drug reactions classified by the Preferred Term (*CICL to medication error means-circumstance or information capable of leading to medication error)

The first three commonly reported ADRs by PT were diarrhoea (14), dizziness (7) and pruritus. Figure 1 shows the frequency of ADRs classified by PTs.

Most of the ADRs started on the same day the suspected drug was administered and, on the average, the ADRs lasted for 3.5 days. All patients who had ADRs fully recovered without sequalae.

### Description of serious ADRs

Of the 53 ICSRs received during the study, 3 (5.7%) were serious (life-threatening) and the rest non-serious. The first serious ICSR was received from a 26-year-old male COVID-19 patient who was given azithromycin and hydroxychloroquine and on the same day (17^th^ April 2020), the patient had supraventricular tachycardia, chest discomfort and abdominal pain which all started the same day the suspected drugs were administered. Concomitant drugs started the same day as the suspected drugs were zinc, enoxaparin injection, vitamin C and ceftriaxone. Hydroxychloroquine and azithromycin were withdrawn on the second and third days respectively with the patient treated with antacids (a suspension of alginic acid, aluminium hydroxide and magnesium trisilicate and esomeprazole).

All other medications were continued until the fifth day (22^nd^ April 2020). On the 9^th^ day (26^th^ April 2020) from the start of the ADRs the patient recovered fully and discharged home.

The second serious ICSR was in a 35-year-old female COVID-19 positive patient, with asthma comorbidity who was given aminophylline and magnesium sulfate on 7^th^ May 2020 for severe life-threatening asthmatic attack and stopped on the same day. On the same day, the patient was given hydroxychloroquine, azithromycin, vitamin C, ceftriaxone, methylprednisolone, salbutamol nebuliser, adrenaline, zinc and enoxaparin. The patient had abdominal pain, headache, sinus tachycardia, dyspnoea, nausea and vomiting, which started on the day these drugs were given. These reactions lasted for 24 hours, although all drugs were continued except adrenaline which was given as a single dose.

The third serious ICSR was as a result of a medication error which occurred in a 47-year old male who was administered IV azithromycin by bolus IV injection instead of by IV infusion. The patient experienced palpitations and nearly ‘passed out’. The reactions stopped when the suspected medication was discontinued.

### Causality Assessment of ADRs

Causality assessment was done for 98 out of the 101 ADRs received with 7(7.1%) and 70(70.1%) classified as certain and possible respectively.

Table 2 shows the detail of the causality assessment terminology assigned to the ADRs.

**Table 2:** Causality scores assigned to the ADRs

WHO-UMC Terminology	Number of ADRs*	Proportion (%)
**Certain**	7	7.1
**Possible/Likely**	70	71.4
**Probable**	10	10.2
**Unlikely**	10	10.2
**Conditional/Unclassified**	0	0.0
**Unassessable/Unclassifiable**	1	1.0

* Causality assessment was done for all ADRs except PTs classified as medication errors and circumstance or information capable of leading to a medication error.

## Discussion

To the best of our knowledge, this is the first study to review ICSRs received from the spontaneous reporting system in the ongoing COVID-19 pandemic. The study identified 3(5.7%) serious (life-threatening) ICSRs, but these were self-limiting, and the patients fully recovered without any sequelae. The low percentage of serious reports was not consistent with what was reported to the VigiBase for which the per cent of serious ADRs was 51.9%.^27^

The mean age of those who experienced ADRs was 37.8 years with the majority, 54.7%, of reports from females. The mean age was consistent with those who tested positive for COVID-19 in Ghana, as reported by the Ghana Health Service.^4^ However, the per cent of males to females who had ADRs is not in line with the per cent of males and females who tested positive for the virus and also in the June 2020 interim report on the descriptive analysis of COVID-19-related spontaneous reports from VigiBase in which more males (55.7%) were reported of having ADRs compared to 38.8% in females.^28^ The slightly higher percentage of ADR reports in females compared to males is however in line with spontaneous ADR reporting rate in Ghana and also as reported by other studies.^29^,^30^,^31^

Analysis of the 95 ADRs which were neither therapeutic failures, medication errors and circumstance or information capable of leading to medication error showed that 85(90.4%) of these were described in the SmPCs of the suspected products, only 9 (9.6%) were not described in the SmPCs. In all the nine cases, there were concomitant treatments, but the ADRs were not expected for the concomitant treatments. It is therefore important that these ADRs are watched closely for possible signals as these medications continue to be used in COVID-19. Additionally, COVID-19 is an emerging pandemic, and new symptoms are being discovered, it may be too early to link these ADRs to the suspected drugs. For instance, the most commonly reported ADRs, namely; diarrhoea (14), dizziness (7), dyspnoea (5) and headache have been reported as symptoms of COVID-19. 32,33

Cardiac related ADRs accounted for 11(10.9%) of all ADRs with hydroxychloroquine alone, hydroxychloroquine in combination with azithromycin and chloroquine alone accounting for 5 (45.5%), 3 (27.3%) and 1(9.0%) respectively. The ADRs reported were consistent with the SmPCs of these products and supported the need for caution with the use of these medications in patients with cardiac-related comorbidities. The majority (70.4%) of the ADRs were rated as possible, indicating that there could be alternative causes of the reactions apart from the suspected drug. Although the study has limitations, as the first study to look at the safety of the medicines issued EUA with a focus on spontaneous reports, it provides some guidance for policy direction. There is a need for the continuous strengthening of the pharmacovigilance Ghana to ensure that the level of underreporting of ADRs is reduced and healthcare professionals at all levels of the healthcare delivery system report ADRs to the FDA. Pharmacovigilance reporting should be included as an indicator in the District Health Information Management System (DHIMS) database to ensure its prioritisation at all levels of the healthcare delivery system.

### Lessons learnt

Lessons learnt during this study were, first of all, the relatively strong spontaneous reporting system and the focal persons at the healthcare facilities and the FDA's regional offices played a major role in the collation of ADR data and information sharing with the Treatment Centres during the pandemic. Secondly, the availability of reporting forms and the Safety Monitoring Plan to inform healthcare workers in the Treatment Centres about the pharmacovigilance requirements during the pandemic made it possible for healthcare professionals to report safety issues. It is hoped that these structures we relied on in this study will be used to monitor in the likely event COVID-19 vaccine is introduced in Ghana.

### Limitations of the study

The first limitation of this study is the small number of ADR reports reviewed; however, this is mainly a preliminary study to present ADRs spontaneously received from healthcare professionals during the pandemic. Secondly, the number of patients who received each of the suspected drugs (i.e. the denominator) was unknown, so we were unable to calculate the rate of ADRs for each medication. Thirdly, underreporting of ADRs in Ghana is known^33^, and the number of reports received may be an underestimate of the real number. We, however, overcame this by constant reminders to healthcare professionals to monitor patients and report all ADRs in patients being treated for COVID-19. The preliminary findings demonstrate that there were no significant safety concerns with the medications issued with the Emergency Use Authorization for the treatment of COVID-19 in Ghana.

## Conclusion

ADR reporting from Treatment Centres was low with top three ADRs being diarrhoea, dizziness and pruritus. The profile of most ADRs reported was consistent with the summary of product characteristics with a few exceptions, such as, tremor for doxycycline; scrotal pain, dyspnoea, gait disturbances and dysgeusia for chloroquine; and dry throat, hyperhidrosis, restlessness and increased frequency of micturition for hydroxychloroquine being new as per summary of product characteristics.

A study with a larger sample size with well-defined denominator is paramount in determining the relative risk of medications in SARS-CoV-2 positive patients and incidence rate of newly reported ADRs.
